# Ultrafast Charge Dynamics in Trap‐Free and Surface‐Trapping Colloidal Quantum Dots

**DOI:** 10.1002/advs.201500088

**Published:** 2015-06-24

**Authors:** Charles T. Smith, Marina A. Leontiadou, Robert Page, Paul O'Brien, David J. Binks

**Affiliations:** ^1^ School of Physics and Astronomy and Photon Science InstituteUniversity of ManchesterManchesterM13 9PLUK; ^2^ School of ChemistryUniversity of ManchesterManchesterM13 9PLUK

**Keywords:** nanocrystals, quantum dots, surface trapping, transient absorption, ultrafast spectroscopy

## Abstract

Ultrafast transient absorption spectroscopy is used to study subnanosecond charge dynamics in CdTe colloidal quantum dots. After treatment with chloride ions, these can become free of surface traps that produce nonradiative recombination. A comparison between these dots and the same dots before treatment enables new insights into the effect of surface trapping on ultrafast charge dynamics. The surface traps typically increase the rate of electron cooling by 70% and introduce a recombination pathway that depopulates the conduction band minimum of single excitons on a subnanosecond timescale, regardless of whether the sample is stirred or flowed. It is also shown that surface trapping significantly reduces the peak bleach obtained for a particular pump fluence, which has important implications for the interpretation of transient absorption data, including the estimation of absorption cross‐sections and multiple exciton generation yields.

## 1 Introduction

Colloidal quantum dots (CQDs) have potential application in a number of different technologies, including light emitting diodes and solar cells.^1^, ^2^ Their appeal rests on both the facile, scalable, and cost‐effective solution‐based methods by which they can be synthesized and processed, and the size tunability of their emission wavelength and absorption edge, which enables rational optimization for particular device designs. CQDs can also exhibit high monodispersity and photoluminescence (PL) quantum yields (QY), and narrow bandwidth emissions.^3^

Many of the processes which are key to the efficacy of CQDs in applications, such as exciton recombination and charge injection and extraction, occur on a nanosecond timescale or faster. The cooling of hot photogenerated charges to the band edge typically takes a few picoseconds, Auger recombination of multiexcitons or trions is characterized by time constants of a few tens or hundreds of picoseconds, while the recombination of single excitons is usually a multiscale process that is complete after a period that ranges from a few nanoseconds to more than a microsecond, depending on the material.^4^, ^5^, ^6^ These processes and their typical timescales are summarized in **Table** **1**. This rich dynamical behavior has been the subject of numerous investigations, a particularly challenging aspect of which is the role of surface traps.

**Table 1:** Summary of the processes affecting the population of the conduction band minimum in CQDs, with their typical timescales

Process	Typical timescale	Ref.
Carrier cooling	Few ps	5
Multiexciton recombination	Tens to hundreds of ps	13
Trion recombination	Tens to hundreds of ps	8, 11, 15, 19
Surface trapping	ps to ns	4, 24
Single exciton recombination	ns to μs	28

The small size of a CQD (typically 2–5 nm) means that a large fraction of its constituent atoms lie on the surface. Here, they may be undercoordinated and possess one or more dangling bonds. The electronic states formed may act as traps for electrons or holes, or both, localizing them at the surface. These traps states are known to mediate nonradiative recombination, reducing the PL QY and lifetime, and enable the formation of trions and PL intermittency, sometimes known as “blinking.”^7^, ^8^, ^9^ Understanding their role and distinguishing their effects from those of other processes is complicated by the sample‐specific contribution of traps to charge dynamics, which can vary, depending on preparation and handling, in ways that are not yet well understood or controlled.

In particular, surface traps can influence charge dynamics on a subnanosecond timescale. The geminate partner of a trapped charge can remain in the volume of the CQD so that a trion is formed when another electron–hole pair is subsequently excited. Trions can recombine by an efficient Auger process with a characteristic lifetime of a few tens or hundreds of picoseconds^4^, ^10^, ^11^, ^12^, ^13^, ^14^ leaving the charge trapped on the surface and one within the CQD volume so that subsequent excitation results in the formation of another trion.^15^ Charges can remain trapped for an extended period; for example a lifetime of 30 s was estimated for a charge trapped on the surface of PbSe CQDs.^15^ This trapping allows a significant population of charges to accumulate if a volume of CQDs is excited repeatedly and the period between excitations is less than this lifetime. Biexcitons also primarily recombine via an Auger process with a similar lifetime to trion recombination,^11^ and this similarity complicates the interpretation of the transient spectroscopic data typically used to determine the efficiency of multiple exciton generation (MEG). In bulk semiconductors, the energy of an absorbed photon in excess of the band gap is typically lost to heat within a few picoseconds,^16^ as the photogenerated carriers rapidly cool to the band edge; this energy loss is a significant limit on the efficiency of solar cells. However, in CQDs an alternative process, MEG, can become efficient in which this excess energy is instead used to generate additional carriers which increase the photocurrent and thus can improve the efficiency of a solar cell.^16^ MEG was first demonstrated a decade ago using ultrafast transient spectroscopy,^17^ but there was initially a discrepancy in the efficiencies reported by different groups.^18^ This problem was subsequently explained by trion recombination resulting from the accumulation of a significant number of surface‐trapped charges. The presence of subnanosecond decay in a photoinduced absorption or PL feature was taken as the signature of biexcitonic recombination, which if it persistent at excitation fluences so low as to have a negligible chance for a CQD to absorb more than one photon per pump pulse, was attributed to MEG. However, since trion formation also requires only the absorption of a single photon per pump pulse and because trion recombination also produces subnanosecond decay, the underlying MEG efficiency was obscured. In 2008, the role of trions was recognized after a number of studies showed that for static samples of CQDs, the effects of trion recombination could become significant.^19^ Fortunately, it was also shown for PbSe CQDs that if the sample was sufficiently stirred or flowed then the resultant refreshing of the excitation volume prevented significant trion formation.^15^, ^19^, ^20^ However, later studies on InAs,^21^ ZnTe/ZnSe,^22^ and HgTe^23^ CQDs showed that for stirred samples, in which the CQDs are eventually recycled through the excitation volume, trion formation was not suppressed at high excitation rates.

Subsequent to the discovery of the role of trion formation, further work revealed that band edge carriers could be trapped to the surface of CQDs on a subnanosecond timescale, resulting in ultrafast spectroscopic decay transients similar in form to those expected to indicate the occurrence of MEG. Tyagi et al.,^24^ compared the decay transients and the pump‐induced absorption change spectrum for freshly synthesized CdSe CQDs and for the same CQDs after extended exposure to high excitation fluences. It was found that the fresh CQDs exhibited a spectrum comprised solely of a band edge bleach, and at low fluences this bleach decayed monoexponentially on a multi nanosecond timescale, consistent with the typical lifetime of single excitons in CQDs. In comparison, the CQDs which had been subjected to the significant beam exposure had an absorption change spectrum that included a broad photo­induced absorption (PA) feature at wavelengths close to and somewhat to the red of the bleach feature. The spectroscopic decay transients associated with these CQDs also changed, becoming multiexponential and more rapid in character. This change in behavior was attributed to an increase in the number of surface states due to the beam exposure, enabling both the PA feature and a rapid depopulation of the band edge seen as the new subnanosecond components to the decay transient. The authors noted in particular that this rapid surface trapping had the potential to obscure MEG efficiency. Subsequent to the observations for CdSe CQDs, similar ultrafast surface‐trapping behavior was reported for ZnTe/ZnSe^22^ and HgTe^23^ CQDs, i.e., a PA feature concurrent with a subnanosecond bleach decay. These observations suggested two criteria that could be used to identify whether ultrafast trapping is prevalent in a sample and thus likely to obscure the assessment of MEG efficiency. A sample suitable for the reliable determination of MEG efficiency should first have no significant broad PA in the pump‐induced absorption spectrum, and second, below the MEG threshold and at moderate excitation fluences, the bleach feature should decay monoexponentially with a lifetime consistent with radiative recombination of a single exciton.

However, all these observations have been made on samples for which there is always a significant amount of surface trapping. For instance, even freshly‐synthesized CdSe CQDs typically exhibit a PL quantum yield (QY) considerably less than 100% because of the nonradiative pathways enabled by unsaturated surface bonds. Until recently, the only CQDs reported to have close to unity QY have been Type I core/shell CQDs.^3^, ^25^ In this structure, the shell acts as a barrier between carriers generated in the core and the surface of the shell; a graduation between core and shell materials is also necessary to ensure that there is no nonradiative recombination caused by an abrupt interface. However, while the shell may act as an effective barrier between surface traps and the carriers at the band edge, MEG studies necessarily involve generating carriers with excess energy above the band edge at least equal to the band gap;^26^ at these energies, the shell band gap may no longer be sufficient to act as a barrier to the surface and the carrier wave function becomes more extended, increasing interaction with the surface in any case. Additionally, thick shells can act as barriers to band edge charge extraction hindering device performance. The presence of traps in all ultrafast spectroscopic studies of CQDs to date raises the possibility that measurements are being affected by them in an as‐yet unrecognized way. However, recently halide treatments of the CQD surface have been developed^27^ which greatly decrease charge trapping without reducing solar cell efficiency.

In this work, the ultrafast charge dynamics in CdTe CQDs are compared before and after a treatment with chloride ions that can completely passivate all surface traps, enabling their effects to be fully determined for the first time. Their influence on the magnitude and dynamics of the pump‐induced absorption change spectrum are examined, and related to the underlying charge dynamics. The implications of these observations for the interpretation of ultrafast transient data are also discussed.

## 2 Results

**Figure 1:**
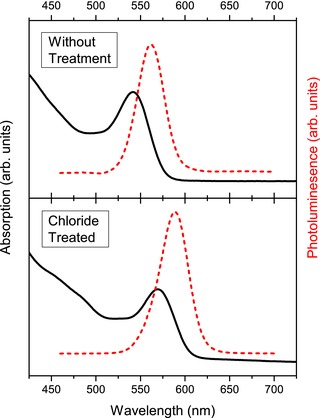
Example of absorption (solid lines) and PL (dashed lines) spectra for a CdTe CQD sample before and after the chloride treatment. The PLQY before and after treatment were 4.4% ± 2.5% and 85.5% ± 2.5%, respectively.

Four independently synthesized CdTe CQD samples were investigated before and after chloride passivation. The undercoordination of Te on the CQD surface before treatment results in the formation of trap sites that mediate nonradiative recombination. The chloride treatment etches and replaces this surface Te, while the Cd bonds to oleylamine.^28^ Representative absorbance and photoluminescence (PL) spectra before and after chloride passivation are shown in **Figure** **1**—similar spectra for the other samples studied are shown in Figure S1 (Supporting Information). Before passivation, the spectral position of the first absorption peak varied from 540 to 600 nm between samples, indicating that the average CQD diameters ranged from 3.2 to 3.6 nm.^29^, ^30^ The shapes of both the absorption and PL spectra are largely unchanged after passivation, except for a 10–30 nm redshift. A previous study^28^ of the effect of the chloride treatment on CdTe QDs has shown that it typically results in a spectral change, which can either be a small blueshift or redshift depending on experimental conditions. Three different effects of the chloride treatment were identified as contributing to the net spectral change. The etch of the surface Te reduced the average size of the CQDs by 0.1 nm^28^ and thus increased the confinement of the carrier wave functions, producing a blueshift. The addition of chloride ions to the CQD surface is also likely to produce a change in confinement potential, and consequent shift in energy of the band edge states. Finally, the treatment was shown to fill trap states near the valence band edge, narrowing the band gap and hence producing a redshift.^28^ The balance of these factors was found to be sensitive to the details of the passivation process, particular the treatment time.^28^ Most notably, the treatment also results in a dramatic increase in PL QY, typically from <10% to ≈90%, but in the best cases reaching unity, to within experimental uncertainty. This observation demonstrates that nonradiative recombination has been eliminated and therefore surface traps have been completely passivated; the PLQY value for each sample studied is given in Table S1 (Supporting Information).

**Figure 2:**
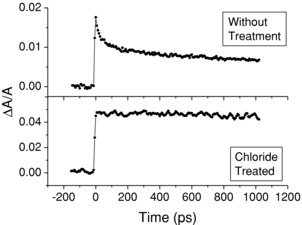
Fractional absorption change, Δ*A*/*A*, transients for a CdTe CQDs sample with (lower) and without (upper) chloride passivation. The samples were pumped at a wavelength of 420 nm and fluence of 1.1 × 10^14^ photons cm^−2^ per pulse, and probed at wavelengths of 598 and 567 nm for the sample with and without passivation, respectively.

For the same excitation conditions, **Figure** **2** compares the fractional change in absorbance produced in a sample of CQDs before and after chloride passivation, both were excited to a level significantly above the conduction band minimum (CBM), but below the MEG threshold. (Similar transients obtained for the other samples are shown in Figure S2 in the Supporting Information.) The sample was probed at a wavelength corresponding to the first peak in the absorption spectrum, and thus monitored the occupation of the CBM of the CQDs.^31^ All the changes in absorbance observed were a reduction, i.e., a bleach, and were thus attributed to state filling of the CBM.^31^ For the untreated samples, a significant and rapid decay in the bleach is observed that can be well described by a biexponential characterized by time constants of about 30 and 500 ps, indicating a rapid depopulation of the CBM. In contrast, no similar decay is observed for the treated sample. The PL decay transients obtained for this type of CQD have been previously reported.^28^ Before passivation, the decay transients are nonmonoexponential in form, with a biexponential or a triexponential function typically required to describe the decay well, and the associated time constants were all >1 ns. This nonexponential form is a consequence of significant trap mediated, nonradiative recombination.^32^ In contrast, after the chloride treatment, the PL transients are all well described by a monoexponential decay with a single characteristic time constant of typically 20 ns. Example PL decays before and after treatment are given in Figure S3 (Supporting Information). The decay constant for the treated samples is a good estimate of the radiative lifetime since they have a PL QY ≈100%, and hence it can be concluded that radiative recombination does not make a significant contribution to the bleach decay over the timescale of the experiments discussed here.

The recombination of biexcitons can produce a subnanosecond decay component in absorption transients similar to the one shown for the untreated sample.^33^ In this experiment, biexcitons can be produced only by the absorption of two photons by a CQD, since the photon energy used is less than twice the band gap, i.e., below the threshold for MEG.^26^ Thus, the contribution of biexciton recombination to the observed decay can be determined by calculating the average band edge occupancy of the CQDs from 〈*N*〉 = *σJ*, where *σ* is the absorption cross‐section at the pump wavelength and *J* is the pump fluence. This expression assumes that every absorbed photon results in an electron relaxing to the CBM. For the experimental conditions used in this study, these calculations yield a maximum value of 〈*N*〉 ≈0.18, which corresponds to a decay in the bleach signal due to the recombination of biexcitons of ≈10% of the maximum amplitude (full details of this calculation are given in the Supporting Information). This is less than the amplitude of noise and so consistent with the transient observed for the treated sample. Conversely, for the untreated sample biexciton recombination cannot account for the large decay component seen, indicating that another process is causing a significant reduction in the bleach.

As detailed in the Introduction section, if the accumulation of trapped charges onto the surface of the CQDs over many pump pulses is an important process in a sample, then trion recombination could produce significant subnanosecond decay in the bleach. In which case, stirring the sample has been shown to decrease the decay of the bleach because it circulates CQDs through the pumped volume, reducing the buildup of surface‐trapped charges and hence of trion formation.^20^ However, for both the untreated and treated CdTe CQDs studied here, no differences in the transients were observed between when the samples were stirred or flowed and when they were held static, under the experimental conditions used (as shown in Figure S4 in the Supporting Information). This shows that the bleach decay observed for the CdTe CQDs before chloride passivation is not primarily due to trion recombination and suggests, as has been shown for other materials,^21^, ^22^, ^24^ that it is instead due to the subnanosecond depopulation of electrons from the CBM to surface states. The absence of this decay after chloride passivation, a surface treatment, confirms the role of the surface in this process, as XPS data has previously shown chloride ions to only reside on the surface of the CQDs.^28^

**Figure 3:**
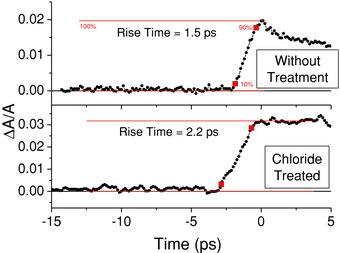
Rise of the fractional absorption change, Δ*A*/*A*, signal for a CdTe CQD sample with and without chloride passivation. The samples were pumped at a wavelength of 420 nm and fluence of 1.1 × 10^14^ photons cm^−2^ per pulse and probed at wavelengths of 598 and 567 nm for the sample with and without passivation, respectively. The rise time is defined as the period for the signal to rise from 10% to 90% of the maximum bleach, as indicated by the square marks; the straight lines indicate the 0% and 100% signal levels.

Higher resolution transients for a sample before and after treatment are shown in **Figure** **3**; similar plots for the other samples are shown in Figure S5 (Supporting Information). As for the lower resolution transients shown in Figure 2 and Figure S2 (Supporting Information), there is no observable decay of the bleach for the treated sample. Further, on this shorter timescale, a significant difference is apparent in the rate of the initial rise of the bleach between the two samples. The mean time for the signal to rise from 10% to 90% of its maximum value for the untreated samples was 1.5 ± 0.1 ps, while for the treated samples it was 2.2 ± 0.1 ps. The corresponding average rates of energy loss were 6.1 ± 0.1 eV ps^−1^ and 3.6 ± 0.2 eV ps^−1^, respectively, i.e., chloride passivation produces a 40% decrease in the cooling rate to the CBM following identical excitation by the pump beam.

In addition to the contrasting decay behavior discussed above, the magnitude of the bleach response was also significantly different after the chloride treatment. For the examples shown in Figures 2 and 3, the maximum bleach for the CQDs before treatment is significantly less than the value seen after chloride passivation even though the pump power is the same in both cases. A similar increase in maximum bleach is also observed for the other samples following passivation with chloride treatment (see the Supporting Information). The pump‐induced absorption change, Δ*A*/*A*, expected for a particular pump fluence can be calculated from the sample absorbance, CBM degeneracy, and absorption cross‐section at the pump wavelength.^23^ This calculation (as detailed in the Supporting Information) results in a good agreement between the expected and experimentally measured values of Δ*A*/*A* for the passivated samples. In contrast, the experimental values obtained before passivation are typically less than the expected value by a factor of 2–3. This suggests that for the untreated samples not every absorbed photon results in an electron at the CBM, i.e., some of the hot electrons created immediately after absorption are cooling directly into a trap state, and thus reducing the CBM state filling responsible for the bleach signal. The surface trapping of hot carriers in CQDs has been discussed at length by Kambhampati^34^ and is also used to explain one form of PL intermittency, also known as “blinking.”^8^, ^35^ This additional cooling pathway for hot electrons in the CQDs before passivation also explains the decreased rise time of the bleach signal, as the population of hot electrons is depleted more rapidly.

**Figure 4:**
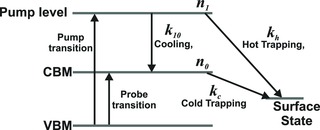
Illustration of the rate of equation model describing the relationship between the hot trapping rate and both the rise time and magnitude of the CBM population.

The quantitative relationship between the enhanced cooling rate and the reduced bleach maximum can be understood using a simple rate equation model, as illustrated schematically in **Figure** **4**. In this model, the pump beam excites electrons from the valance band maximum (VBM) to an initial “pump level” with population, *n*_1_. From this pump level, the hot electrons can cool at a rate characterized by *k*_10_ to the CBM, which has a population, *n*_0_, which is detected by the probe beam. Alternatively, the electrons in the pump level can undergo “hot trapping” directly to a surface state, at a rate characterized by *k*_h_. Electrons can also reach a surface state via “cold trapping” from the CBM, at a rate described by *k*_c_.

The evolution of the population of the CBM, which determines the bleach signal observed experimentally, is thus given by the following coupled rate equations

(1) $${\dot{n}}_{1}\left(t\right)=-\left(k_{h}+k_{10}\right)n_{1}\left(t\right)$$

(2) $${\dot{n}}_{0}\left(t\right)=k_{10}n_{1}\left(t\right)-k_{c}n_{0}\left(t\right)$$

which can be solved to yield

(3) $$n_{0}\left(t\right)=\frac{k_{10}n_{1}\left(0\right)}{k_{10}+k_{h}-k_{c}}\left[e^{-k_{c}t}-e^{-(k_{10}+k_{h})t}\right]$$

The hot trapping rate constant, *k*_h_, appears in both the second exponential term of Equation (3), which determines the bleach rise time, and in the denominator of the prefactor, determining the bleach response. Thus, increasing the value of *k*_h_ simultaneously decreases the rise time and reduces the bleach amplitude. Equation (3) also shows that the CBM of CQDs which experience trapping would completely depopulate on a subnanosecond timescale characterized by $k_{c}^{-1}$. In contrast, a fraction of the bleach remains after ≈1 ns in the experimental transients for the samples before treatment (see Figure 2 and the Supporting Information), and is attributed to a subset of the CQD population that is trap‐free at subnanosecond timescales. As described in the Supporting Information, Equation (3) may be fitted to the transients shown in Figure 3 and Figure S5 (Supporting Information). This yields reliable values for *k*_10_, averaging 1.4 ± 0.1 ps^−1^ for the four samples studied, from the transients for the treated, i.e., trap‐free CQDs, for which *k*_h_ = *k*_c_ = 0. However, fitting to the transients for the untreated samples does not produced well‐constrained results because of the increased number of free parameters.

**Figure 5:**
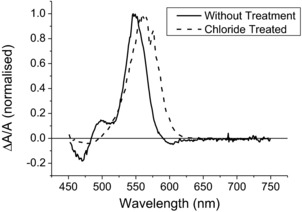
Normalized fractional absorption change, Δ*A*/*A*, spectra for a CdTe CQD sample with and without chloride passivation, collected at a pump–probe delay of ≈1.5 and 2.2 ps, respectively. The samples were pumped at a wavelength of 420 nm pump beams and fluence of 1.1 × 10^14^ photons cm^−2^ per pulse, (pure toluene, the sample diluent, was found to provide no observable contribution to the signal).

Similar picosecond‐scale trapping has been reported previously for a number of CQD types. In the study by Tyagi and Kambhampati,^24^ the authors noted that a significant photoinduced absorption feature developed at the same time as the subnanosecond bleach decay component as surface traps were created on the CdSe CQDs by high beam exposure. This broad feature was close to the band edge and similar PA features in the region of the band edge have been observed for other CQDs, including ZnTe/ZnSe core/shell CQDs.^22^ Example: pump‐induced absorption change spectra for a sample of the CdTe CQDs studied here, before and after chloride treatment, are shown in **Figure** **5**; where experimental conditions are identical to the transients discussed above, and the probe wavelength is scanned rather than pump–probe time delay. The spectra were collected at the pump–probe time delay for which the bleach transient for that sample, as shown in Figure 3 and Figure S5 (Supporting Information), was at a maximum, corresponding to a time when hot excitons have finished cooling to the band edge, but before any significant recombination has occurred. They have been normalized to allow direct comparison of their shapes, and similar spectra for the other samples are shown in Figure S7 (Supporting Information). Each spectrum shows a prominent bleach peak due to state filling of the CBM. Two regions of PA features (negative absorption changes) on either side of the bleach peak are evident for the untreated CQDs. The bleach peak for the untreated samples is always somewhat narrower than that of the corresponding treated sample and also includes a shoulder at about 500 nm. In contrast, the bleach peak for the treated sample is symmetrical, as well as being broader. This suggests that the shoulder is due the supposition of a PA feature onto the short‐wavelength side of the untreated sample's bleach peak. The only PA evident in the treated spectrum is centered at about 475 nm and is much smaller in magnitude than the corresponding feature in the untreated spectrum. These observations are consistent with the almost complete passivation of the CQD surface by the chloride ions, as evidenced by the high PLQY, all but eliminating the trap states responsible for the PA features. Furthermore, the observation of significant PA for the untreated samples supports the attribution of direct surface trapping as the mechanism causing the subnanosecond decay of the bleach transients shown in Figures 2 and 3 and the Supporting Information.

## 3 Discussion

Surface trapping has long been recognized as key in controlling the optical and electronic properties of CQDs.^2^, ^36^ However, understanding the contribution of trapping processes to charge dynamics is made particularly challenging by sample‐to‐sample variation due to the sensitivity of the surface to the details of preparation and handling. In this work, subnanosecond charge dynamics in trap‐free and nearly trap‐free CQDs are studied for the first time. The comparison of these results with those for less well‐passivated CQDs yields new insights into the effect of traps. It has previously been recognized that the effects of trapping could produce subnanosecond decay in the pump‐induced bleach. However, it was thought that these effects could be prevented by flowing or stirring the sample^15^, ^19^, ^20^ or at least easily detected by the presence of a broad photoinduced absorption feature at wavelengths longer than the band edge that is comparable in magnitude to the bleach.^24^ In contrast, we show that large surface‐mediated bleach decays can be found for well‐stirred or flowed samples that exhibit a narrow band edge photoinduced absorption feature with a magnitude of only <5% of the bleach peak. This insight is of particular importance for the interpretation of transient data in studies of MEG. The presence of subnanosecond decays at low excitation rates in well‐stirred samples is used to infer MEG, and the magnitude of the decay feature is used to calculate MEG QY.^16^ Our results show that to avoid trapping obscuring the effects of MEG, it is not sufficient just to stir the sample and to check for a large photo­induced absorption feature. Instead, these results show that it is necessary in addition to show that there is no subnanosecond bleach decay at sufficiently low excitation rates below the MEG threshold. Further, the bleach decay observed at the same low excitation rates but above the MEG threshold should be monoexponential and characterized by the same time constant found when the samples are excited at a rate sufficient to produce the absorption of more than one photon per CQD per pump pulse.

## 4 Conclusion

The direct cooling of hot carriers to trap states in CQDs has been previously recognized,^34^ but its effect has been difficult to assess without trap‐free samples for comparison. Here, by measuring subnanosecond carrier dynamics in samples before and after a highly effective surface passivation treatment which can eliminate trapping, we have been able both to quantify the effect of trapping on the electron cooling rate and also to show that hot carrier trapping can lead to a large reduction in the magnitude of the peak absorption bleach. Furthermore, trapping of both hot electrons and electrons already cooled to the band edge was found to occur regardless of whether the sample was stirred or flowed, which has been previously shown to eliminate the effects of carrier trapping in some types of CQD.^15^, ^19^, ^20^ Hot carrier cooling processes compete with MEG and so their suppression promises to increase the MEG yield. The importance of the reduction in bleach, which we have shown can be quantitatively related to the hot trapping rate, has not been widely recognized previously. It is key to the interpretation of transient absorption data, including that used to determine the absorption cross‐section and infer MEG. The bleach saturates for high pump fluences as the CBM becomes fully occupied, and this can be used to determine *σ* in ultrafast studies including those of MEG.^37^, ^38^, ^39^, ^40^ However, if a significant fraction of hot electrons cool directly to a trap state and hence do not contribute to the bleach then this will increase the fluence required for saturation of the band edge absorption, leading, if unrecognized, to an underestimation of *σ*. This will lead in turn to an underestimation of 〈*N*〉 for the subset of the CQD population that is trap‐free and may result in biexcitonic decay being misinterpreted as being due to MEG rather than being caused by the absorption of more than one photon. Moreover, some studies of MEG^41^ explicitly make use of the assumption that all absorbed photons cool to the band edge in their analysis, making it even more important to determine the extent of hot carrier trapping in the samples under study.

## 5 Experimental Section

Synthesis, chloride treatment, and characterization of the CdTe CQDs is described in detail in our previous work.^28^ Cadmium oxide in tetradecylphosphonic acid (TDPA) in octadecane, and telluride in trioctylphosphine (TOP) were mixed at 290 °C for between 30s and 6 min to obtain nanocrystals ranging from 3.2 to 4.9 nm in diameter. The surfaces of these CQDs were treated with chloride ions by injecting a 0.33 mol dm^−3^ solution of CdCl_2_ in oleylamine containing tetradecylphosphonic acid (TDPA) into CQDs in toluene at 60 °C, and this temperature held for 15 min.

The subnanosecond charge dynamics were studied using a home‐built ultrafast transient absorption spectrometer (UTA) which has been reported previously^23^, ^42^ and so will only be briefly described here. A Ti:Sapphire regenerative amplifier (Spectra‐Physics Spitfire‐Pro) seeded by a mode‐locked Ti:Sapphire oscillator (Spectra‐Physics Tsunami) produces pulses of 100 fs duration and ≈1 mJ energy at a wavelength of ≈800 nm and 1 kHz repetition rate. Using a beam splitter, 95% of this beam is passed to an optical parametric amplifier (OPA, Light Conversion Ltd. TOPAS‐C) with harmonic generating (HG) crystals to produce a pump beam that is tuneable from the infrared to the ultraviolet. The pump beam is passed through a mechanical chopper synchronized to half the pulse repetition rate and then focused onto the sample. A neutral density (ND) filter is used to reduce the time averaged pump power to 1 mW at the sample. The pump beam diameter was 2.2 mm and hence the pump density was 26 mW cm^−2^, which is equivalent to a fluence of 1.1 × 10^14^ photons cm^−2^ per pulse. The remaining 5% of the beam from the amplifier is directed through a computer‐controlled delay stage to vary the arrival time difference between pump and probe pulses at the sample. This beam is reduced to 1 mW using an ND filter then passed through a sapphire plate to produce a white light continuum, which is split to form the probe and reference beams. After the sample, the probe and reference beams are balanced using an ND filter (in the absence of the pump), passed through a spectrometer (Princeton Instruments Acton sp2500i), then onto separate Si photodiodes. Small differences between the probe and reference beam powers produced by pump‐induced changes in the sample transmittance can thus be detected, using a digital lock‐in amplifier (Stanford Research Systems SR830) synchronized to the chopper to improve the signal‐to‐noise ratio. From these changes, we calculate the fractional transmittance change in the sample, Δ*T*/*T*, or, the fractional absorption change, Δ*A*/*A*. Data shown represent averages of 5–10 scans.

The UTA system is equipped with a magnetic stirring system (Thermo Scientific Variomag Mini) capable of stirring the contents of a cuvette at up to 1000 rpm. Alternatively, a home‐built system capable of anaerobically flowing samples up to 250 mL min^−1^ using a peristaltic pump (Stenner SVP1H7) and large reservoir of CQD sample. This removes the possibility of rapid recycling of the photocharged dots which is possible when stirring. In both configurations, as‐synthesized samples were diluted with anhydrous toluene to an absorbance of ≈0.1 at the probe wavelength. No washing was undertaken to reduce the risk of oxidation during an unnecessary processing step, and thus maintain maximum QY.

The steady state absorption and the PL spectra were measured with the use of a Perkin Elmer Lambda 1050 UV/Vis/NIR spectrometer and a Horiba Jobin Yvon Fluorolog model iHR(FL3‐22) spectrofluorometer, respectively.

## Supporting information

As a service to our authors and readers, this journal provides supporting information supplied by the authors. Such materials are peer reviewed and may be re‐organized for online delivery, but are not copy‐edited or typeset. Technical support issues arising from supporting information (other than missing files) should be addressed to the authors.

Supplementary
