# Supplementary material for: Ultrafast Charge Dynamics in Trap‐Free and Surface‐Trapping Colloidal Quantum Dots
Source: Adv Sci (Weinh). 2015 Jun 24;2(10):1500088. doi: 10.1002/advs.201500088 (PMC5115313; doi:10.1002/advs.201500088)
Supplement: Supplementary file 1 — Supplementary [file ADVS-2-0j-s001.pdf]

Copyright WILEY-VCH Verlag GmbH & Co. KGaA, 69469 Weinheim, Germany, 2015.

ADVANCED SCIENCE
Open Access

Supporting Information

for *Adv. Sci.*, DOI: 10.1002/advs.201500088

Ultrafast Charge Dynamics in Trap-Free and Surface-Trapping Colloidal Quantum Dots

*Charles T. Smith, Marina A. Leontiadou, Robert Page, Paul O'Brien, and David J. Binks**

Supporting Information

Ultrafast charge dynamics in trap-free and surface-trapping colloidal quantum dots

*Charles T. Smith[1], Marina A. Leontiadou[1], Robert Page[2], Paul O'Brien[2], and David J. Binks[1]**

Absorption and Photoluminescence spectra

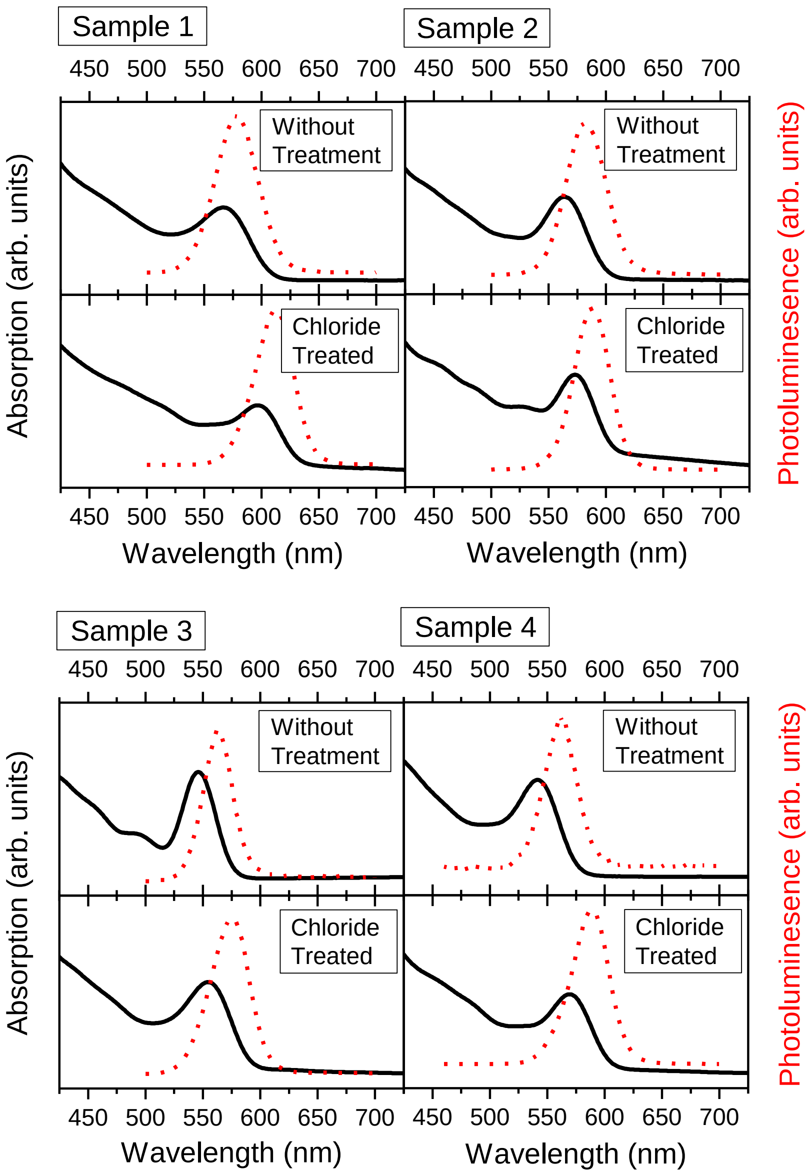

Figure S1. Absorption and PL spectra for the CdTe CQDs before and after chloride treatment, see Table S1 for the peak positions for each sample.

Spectral features and Photoluminescence Quantum Yields

Sample	Band edge transition wavelength (nm)	CQD diameter (nm)	Photoluminescence peak wavelength (nm)	Quantum yield (%) ± 2.5%
1 - Untreated	567	3.4	581	5.3
1 - Treated	598		612	94.8
2 - Untreated	564	3.4	582	10.4
2 - Treated	573		588	99.0
3 - Untreated	547	3.2	564	4.3
3 - Treated	555		575	83.1
4 - Untreated	541	3.1	562	4.4
4 - Treated	570		588	85.5

Table S1. Summary of spectral features and photoluminescence quantum yields for the CdTe CQD samples. The diameters were determined for untreated samples from their band edge transition wavelength using the sizing curves from Yu et al.[1, 2].

Band edge bleach transients

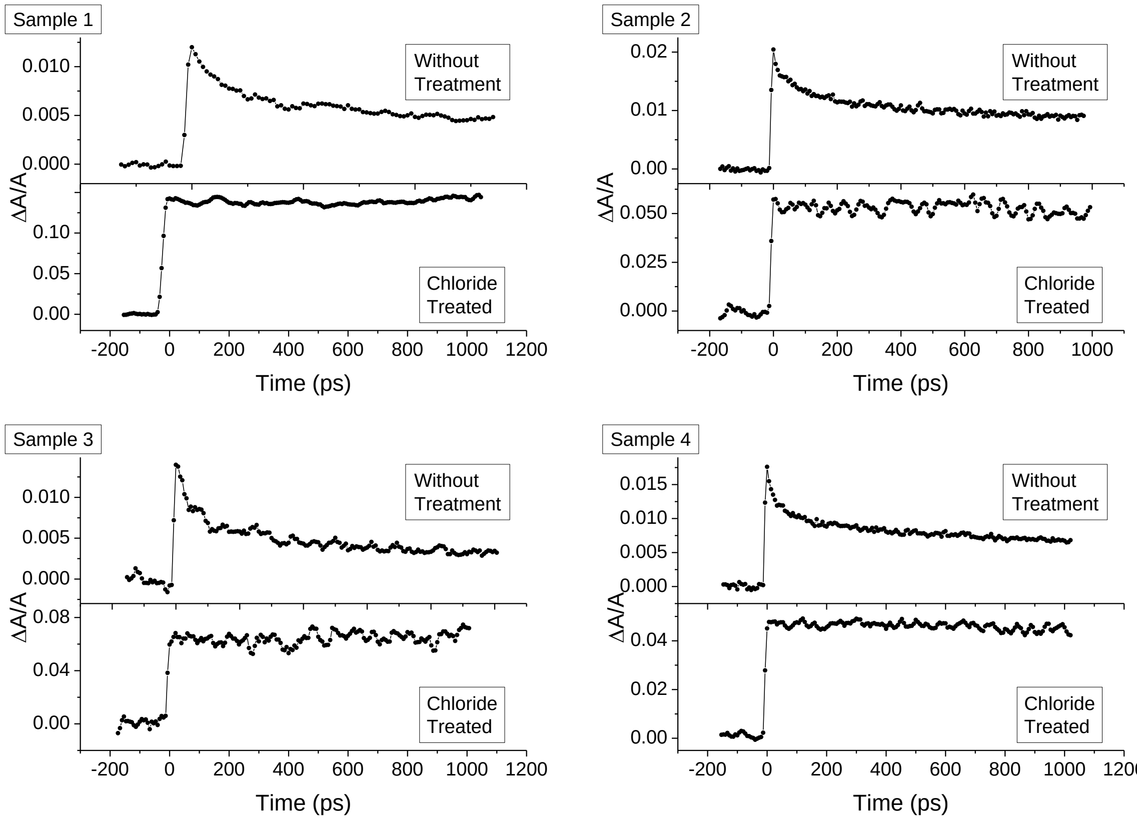

Figure S2. Fractional absorption change, ΔA/A, transients for the CdTe CQDs, with and without chloride passivation. The samples were pumped at a wavelength of 420 nm and fluence of 1.1×10^{14} photons cm^{-2} per pulse, and probed at the wavelength corresponding to their band edge transition, as given in Table S1.

Photoluminescence decay curves

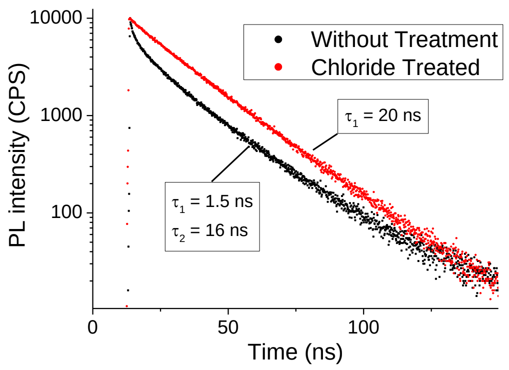

Figure S3. Example PL decay curves for untreated and chloride treated Sample 1, with time constants given from bi-exponential and mono-exponential fits respectively.

Calculation of $\langle N \rangle$ and $\Delta A/A$

The probability of a CQD absorbing N photons per pump pulse, $P(N)$, is determined by Poisson statistics[3]:

$$P(N) = \langle N \rangle^N exp(-\langle N \rangle)/N! \quad \text{(S1)}$$

where $\langle N \rangle$ is the average number of photons absorbed per CQD and depends on the absorption cross-section of the CQD at the pump wavelength, σ_{pump}, and the pump fluence, J (in units of photons per pulse per unit area):

$$\langle N \rangle = \sigma_{pump} J \quad \text{(S2)}$$

Kamal *et al.*[4] found the dependence on diameter, D, of the extinction coefficient for a wavelength of 410 nm, ε_{410}, to be:

$$\varepsilon_{410} = 10600(D)^3 \quad \text{(S3)}$$

where ε_{410} is related to the cross-section at 410 nm, σ_{410}, (in units of cm^{-2}) by:

$$\sigma_{410} = 1000 \ln(10)\, \varepsilon_{410}/N_A \quad \text{(S4)}$$

where N_A is Avogado's constant. $\sigma_{pump}$is found from this by scaling by the ratio of the sample absorbance at the pump wavelength, A_{pump}, and at 410 nm, A_{410}, i.e.

$$\sigma_{pump} = \sigma_{410} \frac{A_{pump}}{A_{410}} \quad \text{(S5)}$$

Once calculated, $\langle N \rangle$ can then be used to estimate the expected value of the maximum fractional absorption change at the band edge, $\Delta A_{max}/A$ [5, 6]:

$$\langle N \rangle = 2 \frac{\Delta A_{max}}{A} \frac{A_{pump}}{1 - exp(-A_{pump})} \quad \text{(S6)}$$

where A is the sample absorbance at the band edge.

The experimentally determined and calculated expected values of $\Delta A_{max}/A$ for the treated samples were consistent, where on average they were different by a factor of 1.05 ± 0.03. For the untreated samples the experimental values were consistently lower, on average by a factor of 0.32 ± 0.01.

If a single CQD absorbs greater than one photon per pump pulse a multi-exciton will be formed, and thus exhibit a fast decay component in transient absorption to a plateau on a picosecond time scale, leaving only single excitons. $\langle N \rangle$ can be used to calculate the expected ratio, $R(J)$, of the peak-to-plateau amplitude[7]:

$$R(J) = \sigma J[1 - \exp(-\sigma J)]^{-1} \quad \text{(S7)}$$

Sample specific values for the observed and expected transient properties for each CQD sample are given in Table S2.

Sample	J (photons cm^{-2} per pulse)	$\langle N\rangle_{pre}$	$\langle N\rangle_{obs}$	$\left(\frac{\Delta A_{max}}{A}\right)_{pre}$	$\left(\frac{\Delta A_{max}}{A}\right)_{obs}$	Signal noise (%)	R_{pre}	R_{obs}
1 Untreated	1.1×10^{14}	0.139	0.027	0.060	0.0134	14.6	1.07	3.18
	8.5×10^{13}	0.111	0.017	0.048	0.0084	12.5	1.06	3.04
	6.4×10^{13}	0.083	0.011	0.036	0.0053	17.3	1.04	4.32
	1.1×10^{14}	0.139	0.054	0.060	0.022	6.0	1.07	>1.72
1 Treated	1.1×10^{14}	0.179	0.155	0.076	0.0636	15.2	1.10	1.00
	8.5×10^{13}	0.143	0.117	0.061	0.0587	11.0	1.08	1.00
	6.4×10^{13}	0.107	0.101	0.046	0.0504	13.8	1.06	1.00
	1.1×10^{14}	0.178	0.369	0.076	0.151	6.0	1.10	1.00
2 Untreated	1.2×10^{14}	0.147	0.041	0.070	0.0204	8.6	1.08	2.28
	8.8×10^{13}	0.110	0.026	0.053	0.0131	9.3	1.06	2.16
	6.4×10^{13}	0.080	0.012	0.038	0.0061	27.6	1.04	2.29
	1.1×10^{14}	0.147	0.052	0.070	0.024	8.5	1.08	>1.36
2 Treated	1.2×10^{14}	0.167	0.115	0.077	0.0577	11.4	1.09	1.00
	8.1×10^{13}	0.113	0.095	0.052	0.0474	15.1	1.06	1.00
	7.5×10^{12}	0.104	0.096	0.048	0.0482	8.7	1.06	1.00
	1.1×10^{14}	0.167	0.135	0.077	0.061	14.1	1.09	1.00
3 Untreated	1.1×10^{14}	0.123	0.028	0.052	0.0140	10.1	1.07	4.20
	1.1×10^{14}	0.123	0.028	0.052	0.011	14.9	1.07	>1.07
3 Treated	1.1×10^{14}	0.127	0.137	0.054	0.0683	28.6	1.07	1.00
	1.1×10^{14}	0.127	0.122	0.054	0.050	19.8	1.07	1.00
4 Untreated	1.1×10^{14}	0.111	0.035	0.051	0.0176	5.0	1.06	2.60
	1.2×10^{14}	0.111	0.045	0.051	0.020	14.3	1.06	>1.53
4 Treated	1.1×10^{14}	0.144	0.095	0.059	0.0477	11.6	1.08	1.00
	8.5×10^{13}	0.115	0.086	0.047	0.0429	7.6	1.06	1.00
	1.2×10^{14}	0.144	0.080	0.059	0.031	14.7	1.08	1.00

Table S2. Predicted (pre) and observed (obs) values for the average band edge occupancy, $\langle N\rangle$, maximum fractional absorbance change, $\Delta A_{max}/A$, signal noise, and peak-to-plateau ratio, R, for each of the four samples before and after treatment. Predicted values are produced by setting J in Equation 2, 6 & 7. Observed values are derived from the same equations but use experimentally measured values of $\Delta A_{max}/A$. Each row of data for a particular sample corresponds to a different pump fluence, J. For transient data taken with a high resolution, the plateau of the transient is not observed and so the observed R is given as a lower limit.

There are numerous key observations from this table, firstly, the observed values for $\langle N\rangle$ and $\Delta A_{max}/A$ are largely in line with the expected values for treated samples, but are significantly lower for the untreated samples. Secondly, a reduction in J does not reduce the observed R values for the untreated samples. And thirdly, the difference between the observed and predicted R values are always greatly in excess of the noise level for the untreated samples, but below for the treated samples. These observations allow us to conclude that the lack of passivation in the untreated sample causes both a decrease in the peak amplitude and a fast decay to a plateau which are not explained by two-photon absorption.

Comparison of stirred or flowed samples with static samples

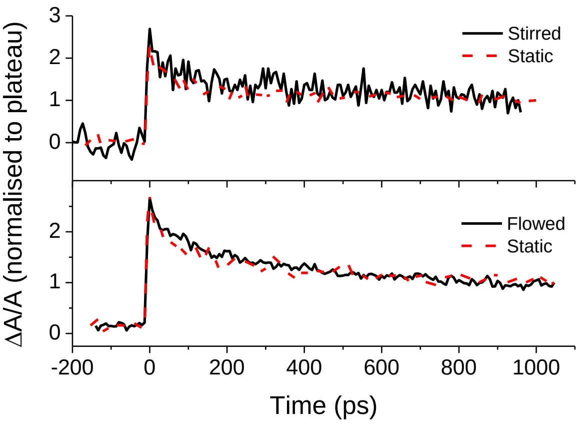

Figure S4. Comparison of fractional absorption change, ΔA/A, transients for untreated CdTe CQDs when the sample is (upper) stirred at 1000 rpm and held static, and (lower) flowed at 250 ml min^{-1}and held static. The samples were pumped at a wavelength of 420 nm and fluence of 1.1×10^{14} photons cm^{-2} per pulse. The stirred and flowed samples were probed at wavelengths of 564 nm and 588 nm, respectively-.

High-resolution band edge bleach transients

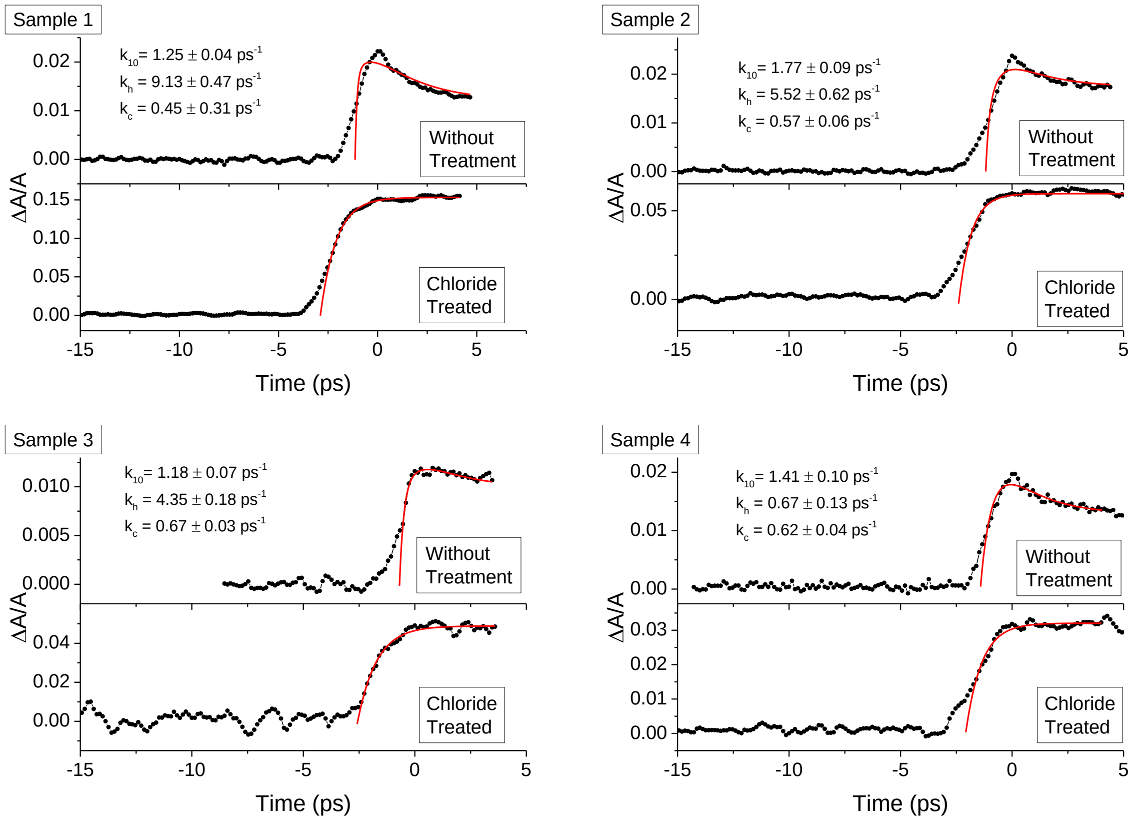

Figure S5. High-resolution fractional absorption change, ΔA/A, transients showing the rise in signal for the CdTe CQDs, with and without chloride passivation. The samples were pumped at a wavelength of 420 nm and fluence of 1.1×10^{14} photons cm^{-2} per pulse, and probed at the wavelength corresponding to their band edge transition, as given in Table S1. The red lines are fits to equation (S8) and equation (S9) for the treated and untreated samples, respectively.

Equation (3) from the main manuscript was fitted to the high resolution transients described in the previous section. The treated samples were assumed to be trap-free and so for fitting to the corresponding transients the rates of the trapping processes were set to zero i.e. $k_h = k_c = 0$ which yields a simple exponential rise

$$n_0(t) = n_1(0)[1 - e^{-k_{10}t}] \quad \text{(S8)}$$

that, as shown in Figure S5, described the transients well, with a only small deviation over the first ~1 ps. The values of k_{10} thus extracted were then used in the fitting of the corresponding untreated samples. The transients for untreated samples shown in Figures 2 and S2 do not decay to zero over the time period of the experiment but rather decay to a plateau. This indicates that there is a fraction of the CQDs that are trap-free in these samples, since trapping completely depopulates the band edge on this time-scale reducing the bleach to zero. Hence, the following equation, which combines terms for the trap-free, n_1^*, and trapping CQD populations, n_1^{tr}, was fitted to the bleach transients for the treated samples:

$$n_0(t) = n_1^*(0)[1 - e^{-k_{10}t}] + \frac{k_{10}\boldsymbol{n_1^{tr}}(0)}{k_{10}+k_h-k_c}\left[e^{-k_c t} - e^{-(k_{10}+k_h)t}\right] \quad \text{(S9).}$$

However, whilst parameters sets could be found that reproduced the observed dynamic (again except for the first ~1 ps of the rise), as shown in Figure S5, the increased number of free parameters, i.e. k_h, k_c, n_1^* and n_1^{tr}, meant that fitting did not yield well-constrained values for these parameters.

The agreement between equation (S8) and the transients for the treated samples is remarkable given the simplicity of the model used. A more sophisticated model, for instance incorporating a more detailed energy level structure, could be constructed which might produce a better match with the data during the first ps of the rise. However, such a model would include a greater number of free parameters which might, as was the case for equation (S9), result in the values yielded by the fit becoming ill-constrained.

Pump-induced absorption change spectra

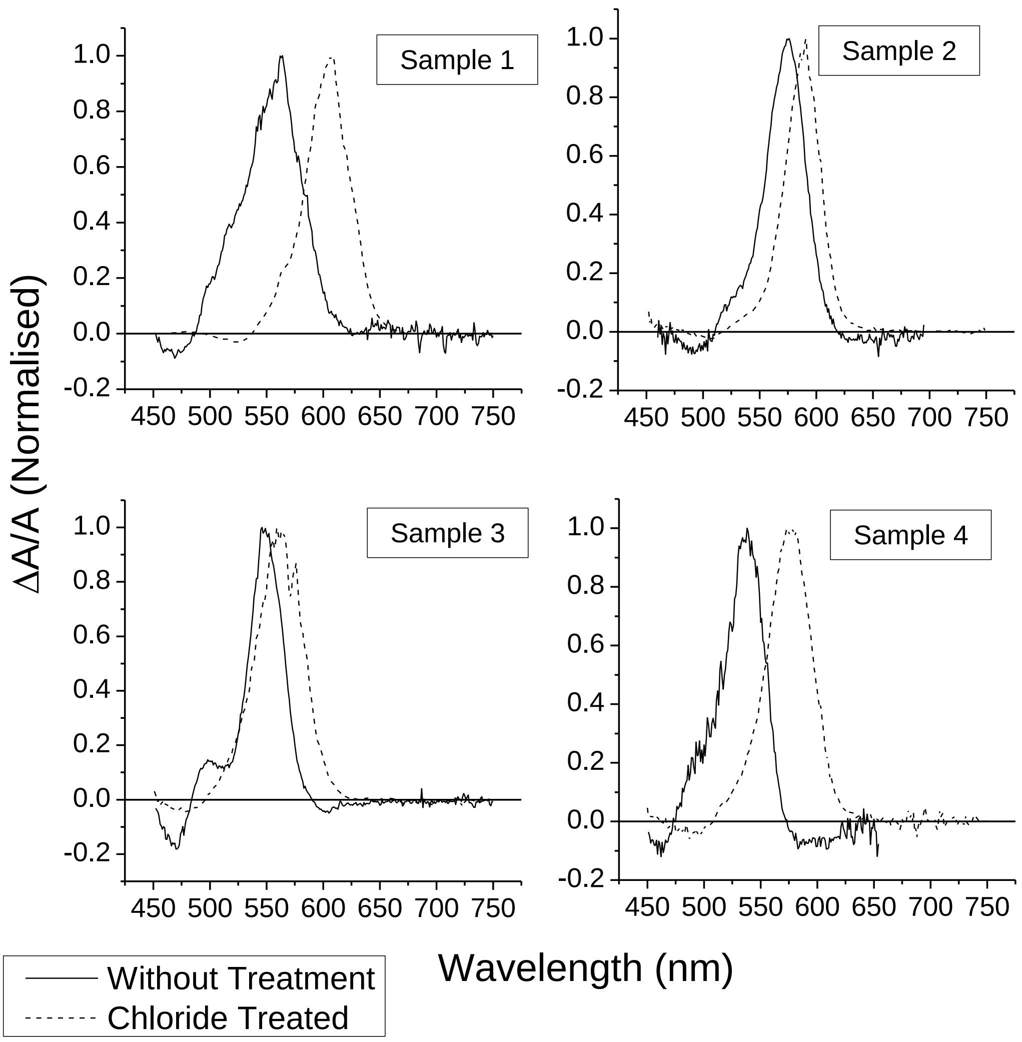

Figure S6. Normalised fractional absorption change, ΔA/A, spectra for CdTe CQD samples with and without chloride passivation. These spectra were collected at a pump-probe delay of between 1.5 ps and 2.5 ps, corresponding to when the bleach transients shown in Figure S5 peaked. The samples were pumped at a wavelength of 420 nm pump beams and fluence of 1.1×10^{14} photons cm^{-2} per pulse, (pure toluene, the sample diluent, was found to provide no observable contribution to the signal).

References

[1] W. W. Yu, L. H. Qu, W. Z. Guo, X. G. Peng, *Chem Mater* **2003,** *15* (14), 2854-2860. DOI 10.1021/Cm034081k.

[2] W. W. Yu, L. H. Qu, W. Z. Guo, X. G. Peng, *Chem Mater* **2004,** *16* (3), 560-560. DOI 10.1021/Cm033007z.

[3] M. C. Beard, *J Phys Chem Lett* **2011,** *2* (11), 1282-1288. DOI 10.1021/Jz200166y.

[4] J. S. Kamal, A. Omari, K. Van Hoecke, Q. Zhao, A. Vantomme, F. Vanhaecke, R. K. Capek, Z. Hens, *Journal of Physical Chemistry C* **2012,** *116* (8), 5049-5054. DOI 10.1021/Jp212281m.

[5] M. Cadirci, S. K. Stubbs, S. J. O. Hardman, O. Masala, G. Allan, C. Delerue, N. Pickett, D. J. Binks, *Phys. Chem. Chem. Phys.* **2012,** *14* (43), 15166-15172. DOI 10.1039/C2cp42125a.

[6] V. I. Klimov, *NANOCRYSTAL QUANTUM DOTS*. CRC Press: **2010**.

[7] M. C. Beard, K. P. Knutsen, P. Yu, J. M. Luther, Q. Song, W. K. Metzger, R. J. Ellingson, A. J. Nozik, *Nano Lett* **2007,** *7* (8), 2506-2512. DOI 10.1021/nl071486l.
